# High-throughput screening and classification of chemicals and their effects on neuronal gene expression using RASL-seq

**DOI:** 10.1038/s41598-019-39016-5

**Published:** 2019-03-14

**Authors:** Jeremy M. Simon, Smita R. Paranjape, Justin M. Wolter, Gabriela Salazar, Mark J. Zylka

**Affiliations:** 1 Department of Cell Biology and Physiology, UNC Neuroscience Center, The University of North Carolina at Chapel Hill, Chapel Hill, NC 27599 USA; 2 Carolina Institute for Developmental Disabilities, The University of North Carolina at Chapel Hill, Chapel Hill, NC 27599 USA; 3 Department of Genetics, The University of North Carolina at Chapel Hill, Chapel Hill, NC 27599 USA; 4 Lineberger Comprehensive Cancer Center, The University of North Carolina at Chapel Hill, Chapel Hill, NC 27599 USA

## Abstract

We previously used RNA-seq to identify chemicals whose effects on neuronal gene expression mimicked transcriptional signatures of autism, aging, and neurodegeneration. However, this approach was costly and time consuming, which limited our study to testing a single chemical concentration on mixed sex cortical neuron cultures. Here, we adapted a targeted transcriptomic method (RASL-seq, similar to TempO-seq) to interrogate changes in expression of a set of 56 signature genes in response to a library of 350 chemicals and chemical mixtures at four concentrations in male and female mouse neuronal cultures. This enabled us to replicate and expand our previous classifications, and show that transcriptional responses were largely equivalent between sexes. Overall, we found that RASL-seq can be used to accelerate the pace at which chemicals and mixtures that transcriptionally mimic autism and other neuropsychiatric diseases can be identified, and provides a cost-effective way to quantify gene expression with a panel of marker genes.

## Introduction

Environmental chemicals have been epidemiologically linked to autism and other neuropsychiatric diseases. Epidemiological studies have linked proximity to the use of certain agricultural pesticides with autism^1,2^, and exposure to a class of insecticides (pyrethroids) has been linked to attention deficit hyperactivity disorder (ADHD) risk^3,4^. How pre- or postnatal exposure to certain drugs or chemicals augments autism risk remains largely unknown, however, there is a significant non-genetic component to risk estimated from heritability studies (10–50%^5–7^), and may be underestimated since these studies do not account for changing environmental influences impacting the population^8^. Given that a small proportion of the patient population has recurrent single gene mutations impacting autism risk^5^, it is important to investigate potential environmental risk factors in addition to exploring the consequence of genetic mutations.

We previously tested how nearly 300 environmental-use chemicals affected gene expression in primary cortical neuron cultures using RNA-seq^9^. We identified a group of chemicals that induced transcriptional changes similar to those observed in autism, aging, and neurodegeneration. This group included rotenone, a pesticide associated with Parkinson’s disease risk^10,11^, and certain fungicides that inhibit mitochondrial complex III, including fenamidone, famoxidone, and the strobilurin fungicides pyraclostrobin and trifloxystrobin. We further showed that previous strobilurin toxicity studies might underestimate exposure risk^12^. Our transcriptional study was limited, however, in that each chemical was tested at only one concentration on mixed sex (male and female) neuronal cultures. As a result, some chemicals that we hypothesized would induce transcriptionally similar responses failed to cluster as expected, in part due to testing at concentrations that were too low. One example of this was azoxystrobin, which induced reactive oxygen species (like other strobilurin fungicides) at a higher concentration than that assayed by RNA-seq. These data suggested a need for a more cost-effective way to profile gene expression across several chemical concentrations, especially when the active concentration of a chemical is not known *a priori*, as was the case in our study. Moreover, males are at greater risk for developing autism than females (~3:1 ratio)^13^, so we also sought a more cost-effective way to profile gene expression so that male-only and female-only cultures could be examined in parallel, to assess whether chemicals had greater effects on one sex.

Targeted transcriptomic approaches, such as RASL-seq^14,15^ and the related technology TempO-seq^16^, are scalable such that screening hundreds of chemicals at multiple concentrations becomes feasible. RASL-seq is performed in 384-well plates and can be automated, which greatly simplifies sample preparation, reduces reagent and drug costs, and allows massive multiplexing to facilitate screening large drug libraries. We sought to adapt the RASL-seq method for use with primary neurons, a traditionally challenging cell type to utilize for high-throughput screens. To apply RASL-seq to neuronal cultures, we designed a probe set that targeted gene markers of neuroinflammation, synaptic function, stress response, and transcriptional signatures associated with autism and neuropsychiatric diseases. This probe set was based on genes and pathways that uniquely marked the six chemical clusters we identified in our prior study^9^. We treated male and female primary neuronal cultures with environmental-use chemicals and chemical mixtures at four concentrations and in each sex (9,216 experiments in total). We found that many of the chemicals identified induced transcriptional responses consistent with that previously shown regardless of concentration, whereas others exhibited varying degrees of concentration-dependence. We further refined our previous chemical classifications based on this new dimension of dosage, and identified new environmental chemicals and mixtures that transcriptionally mimic autism and other brain disorders.

## Results

### Targeted transcriptomics enables screening of chemicals on cortical neuron cultures in a rapid and cost-effective manner

**Figure 1:**
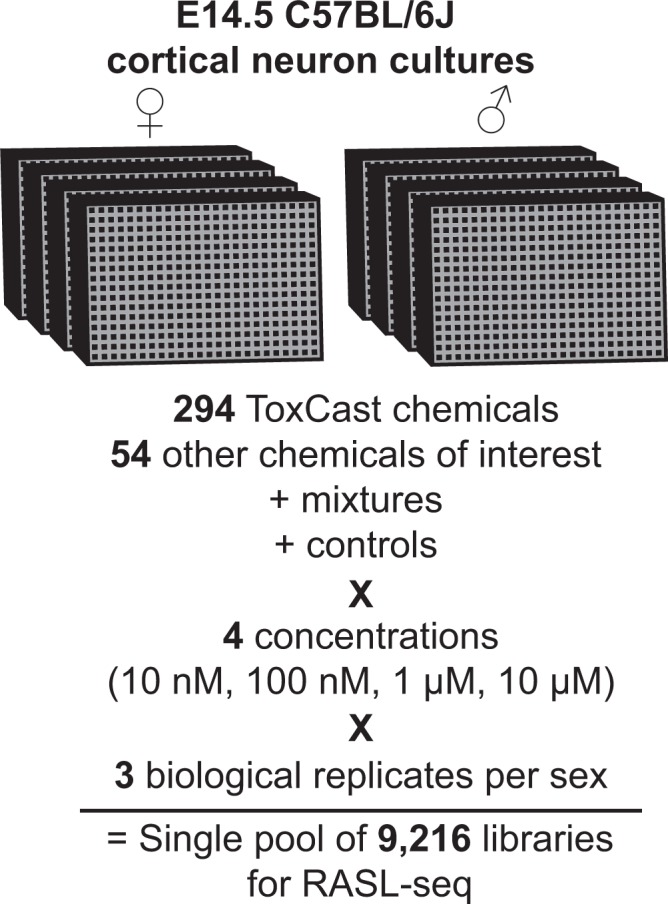
RASL-seq workflow. Sex-specific cortical neuron cultures from E14.5 C57BL/6J mice were plated at a density of 20,000 cells per well in 384-well plates; four plates contained cells from male embryos and four plates contained cells from female embryos. The plates were dosed with 294 ToxCast Phase I chemicals, 54 other commonly used chemicals, chemical mixtures, and controls at four concentrations per sex, in biological triplicate, using liquid-handling robotics. Certain chemicals of interest were also assayed multiple times (Supplementary Data S5). We were able to process a single biological replicate (4 male, 4 female plates at 4 concentrations) in 5–6 h with a liquid handling robot. The 9,216 samples were uniquely barcoded, and then were pooled together and sequenced.

To expand our findings on the transcriptional signatures of environmental use chemicals, we adapted the RNA annealing, selection, and ligation (RASL-seq) assay as previously described^14^ (Fig. 1, Supplementary Fig. S1). We utilized mouse neuronal cultures, which we previously found contain the principle cell types of the brain and models the gene expression signatures of the embryonic/fetal mouse and human brain^9^. We treated male and female mouse neuronal cultures with the entire ToxCast Phase I library (294 chemicals), other chemicals of interest, as well as chemical mixtures, across four concentrations (10 nM, 100 nM, 1 µM, 10 µM), in biological triplicate (Supplementary Data S1–4). These 9,216 samples were processed in twenty-four 384-well plates using liquid-handling robotics such that the entire process was completed in less than 6 hours per biological replicate at a total cost of roughly $2 per sample (including sequencing costs). We designed probes to interrogate expression of 56 genes. These genes were selected based on their responses to chemical clusters we defined previously^9^, including markers of neuroinflammation (e.g. *Cx3cr1*, *Aqp4*), the cytoskeleton (e.g. *Gsn*, *Pdlim2*), stress response (e.g. *Hmox1, Gsta4*), immediate early genes (e.g. *Fos*, *Vgf*), and synaptic function (e.g. *Nrxn1*, *Syt1*), as well as markers of chrX (*Xist*) and chrY (*Ddx3y*), and control genes (*Ascl3*, *Psmd4*, *Sdha*, *Tbp*). We also included a luciferase spike-in and corresponding luciferase gene probes in each well to control for amplification efficiency and cell density.

### High-throughput screen of 350 chemicals and chemical mixtures in cortical neurons

**Figure 2:**
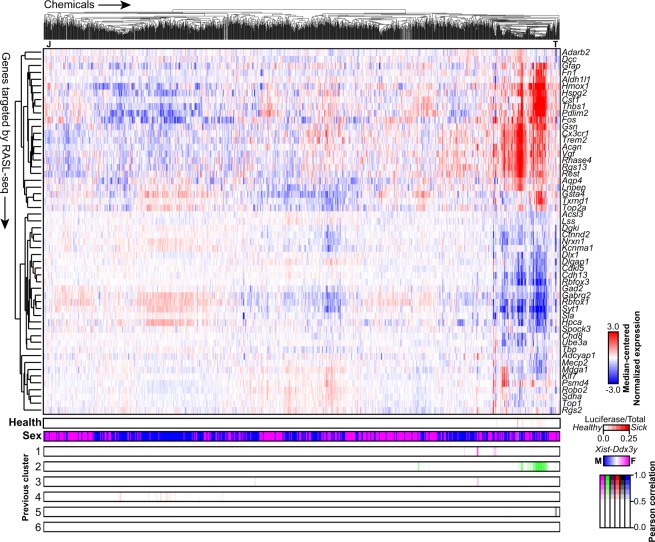
Targeted transcriptional screen identifies chemicals that alter neuronal gene expression. Median-centered normalized gene expression values for all chemicals, concentrations, and sexes (2,791 passed quality control filters) were hierarchically clustered across the 56 genes interrogated by RASL-seq. Biological replicates were averaged. Cell health (bottom) was estimated by assessing the ratio of luciferase spike-in reads to total reads for each chemical; color scale is white (0) to red (0.25). Sex was confirmed by subtracting *Ddx3y* expression from that of *Xist*. Pearson correlation between each sample and aggregated profiles of each of the six chemical clusters identified previously were computed and plotted. (+/−) JQ1, J; topoisomerase inhibitors, T. Full normalized data table provided in Supplementary Data S7.

We first verified that a consistently high percentage of reads mapped correctly in each plate that we processed (Supplementary Table S1). We then filtered the raw data (Supplementary Data S5) by requiring at least 500 reads per well and 1000 reads per probe (summed across all 9,216 samples) to ensure we analyzed data from wells and probes that amplified successfully. We also quantified the luciferase spike-in values relative to the total read count per well to use as a proxy for cell health (Supplementary Fig. S2, Data S6). We then employed a median-polish procedure^17^ and collapsed biological replicates by taking the median (Supplementary Data S7; see Methods). Using these normalized expression values for each gene, we performed unsupervised hierarchical clustering of all chemicals, chemical mixtures, and controls across all concentrations and sexes (Fig. 2). We found numerous robust expression effects, including a large set of chemicals that exhibited an upregulation of genes such as *Hmox1*, *Fos*, *Cx3cr1*, and *Gsta4*, and downregulation of *Rbfox3* (NeuN), *Syt1*, and *Nrxn1*, among others. These expression effects were consistent with the upregulation of immune/cytoskeletal genes and downregulation of synaptic genes that we and others have linked to the transcriptional signature of autism^9,18,19^. A class of known and experimental topoisomerase inhibitors also clustered together and demonstrated downregulation of long synaptic genes (Fig. 2, “T”), as we previously described^9,20^. We also discovered one novel expression cluster, which was comprised of multiple concentrations of one chemical: (+/−) JQ1 (Fig. 2, “J”). (+/−) JQ1 treatment resulted in the downregulation of microglial/astrocytic markers (e.g. *Cx3cr1*, *Trem2*, *Aqp4*), and upregulation of *Fos*, *Rest*, and *Nrxn1*, among others. This unique transcriptional response was somewhat expected, since this chemical is an epigenetic modifier^21^, and therefore has a unique function and purpose relative to the predominantly environmental-use chemicals in this library. The observed downregulation of microglial markers is also consistent with previous reports showing that (+/−) JQ1 reduces neuroinflammation and expression of pro-inflammatory modulators in mouse models of Alzheimer’s disease^22^.

**Figure 3:**
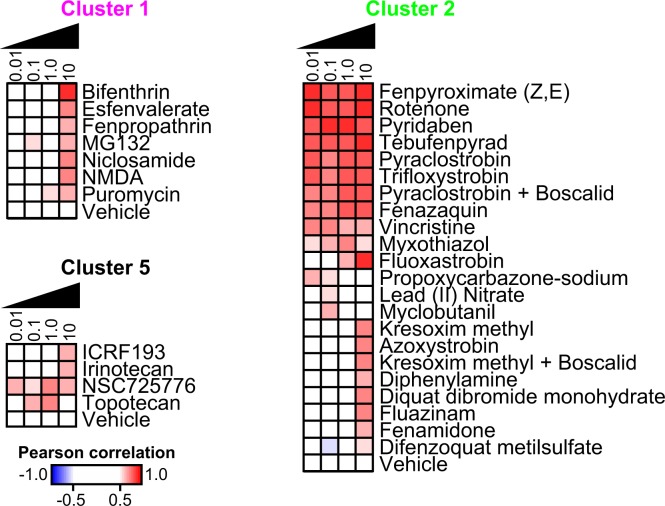
Chemicals and mixtures that recapitulate previously identified transcriptional signatures. Pearson correlations for each chemical and concentration (µM) that corresponded to previously-identified chemicals in Clusters 1, 2, and 5. Absolute value Pearson correlations exceeding 0.5 were colored on a scale of white to blue (−0.5 to −1.0) or white to red (0.5 to 1.0). Biological replicates and sexes were averaged.

To further evaluate how similar these observed expression patterns were to our previously identified chemical clusters that were defined based on whole-transcriptome RNA-seq^9^, we computed Pearson correlations for each chemical-concentration-sex against the aggregated signature of each of the six previous clusters (Fig. 2, bottom). We found that the RNA-seq expression patterns exhibited by chemicals in Clusters 1, 2, and 5 could be strongly recapitulated with RASL-seq data from many of the same chemicals as well as new chemicals, concentrations, and mixtures not tested previously (Pearson r > 0.65; Fig. 3). For example, strobilurin fungicides (including pyraclostrobin, trifloxystrobin) and other chemicals (including fenamidone, fenpyroximate, rotenone), all of which target mitochondrial Complex I or III of the electron transport chain, were previously assigned to Cluster 2 (autism- and neurodegeneration-like signature). These same chemicals clustered together using RASL-seq (Fig. 3).

RASL-seq experiments also identified additional compounds that grouped with Cluster 2 chemicals at certain concentrations. We previously showed that the strobilurin fungicide azoxystrobin induced reactive oxygen species at 10 µM but appeared to be transcriptionally inactive at 1 µM^9^. Using RASL-seq with transcriptional information at multiple concentrations, we now show that azoxystrobin induces gene expression changes consistent with other strobilurin fungicides at 10 µM (Fig. 3). Additional chemicals that induced changes consistent with Cluster 2 included tebunfenpyrad, diquat dibromide, fenazaquin, fluazinam, diphenylamine, difenzoquat metilsulfate, myxothiazol, vincristine, and kresoxim-methyl, as well as mixtures of either pyraclostrobin or kresoxim-methyl with boscalid, each of which have a mechanism of action related to mitochondrial electron transport, oxidative phosphorylation, and/or microtubule destabilization^23–28^. RASL-seq also recapitulated the expression profiles for topoisomerase inhibitors, which downregulate expression of long synaptic genes^9,20^ (Fig. 3). Additionally, we refined the Cluster 1-like chemicals to include pyrethroids bifenthrin, fenpropathrin, and esfenvalerate, as well as NMDA; these chemicals collectively induce expression of immediate early genes (Fig. 2), and have been linked to neurological conditions such as ADHD^3,4^, autism^2^, and Parkinson’s disease^29^.

**Figure 4:**
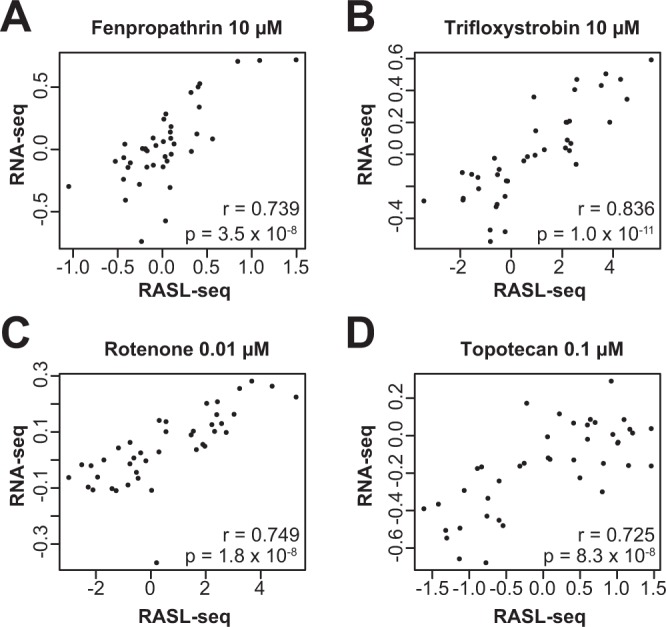
Concordance between RNA-seq and RASL-seq gene expression. Scatterplots of normalized expression values obtained from RASL-seq (this study) and RNA-seq for matched chemical-concentrations of representative chemicals (**A**) fenpropathrin 10 µM (Cluster 1), (**B**) trifloxystrobin 10 µM (Cluster 2), (**C**) rotenone 0.01 µM (Cluster 2), and (**D**) topotecan 0.1 µM (relative to 0.3 µM topotecan assayed by RNA-seq; Cluster 5). Each point is a gene commonly assayed by both studies. Expression values for RNA-seq represent batch-corrected, quantile-normalized, and median-centered data as described previously. Expression values for RASL-seq represent the median-polished values normalized to control genes described here. Pearson correlations and associated p-values are provided.

Further, we directly compared normalized expression values obtained from this targeted transcriptomic approach to those obtained from whole-transcriptome RNA-seq to assess reproducibility across platforms. For hallmark chemicals fenpropathrin (Cluster 1), trifloxystrobin (Cluster 2), rotenone (Cluster 2), and topotecan (Cluster 5), focusing on the genes assayed by both platforms, we observed Pearson correlations greater than 0.72 for matched chemicals and concentrations between this study and our previous study (Fig. 4). We additionally computed pairwise Pearson correlations among biological triplicates of representative chemicals assayed by RASL-seq and found that the measured effects were largely reproducible across replicates, with most Pearson correlations exceeding 0.7 (Supplementary Fig. S3). Together, these data suggest that many of the gene expression effects detected from whole-transcriptome RNA-seq were robust and could be confirmed by an analogous targeted approach.

### Assessment of differences in neuronal response to chemical exposure due to sex

**Figure 5:**
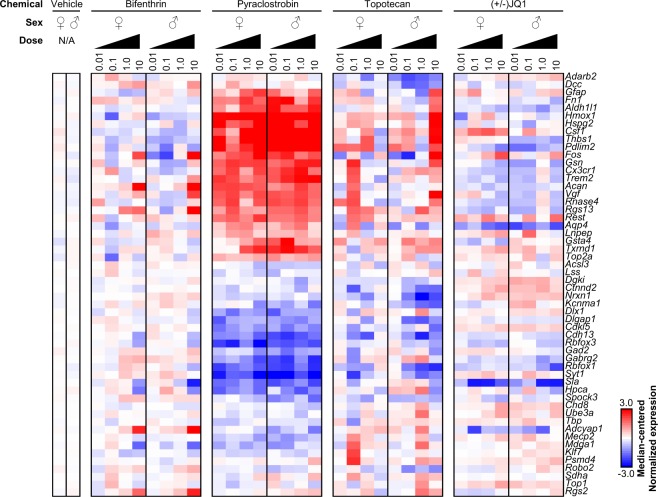
Comparisons of gene expression changes induced by chemicals in male and female cultures. Normalized gene expression values for bifenthrin, pyraclostrobin, topotecan, (+/−) JQ1, and vehicle control were plotted for all concentrations and sexes.

Lastly, we explored whether transcriptional responses were influenced by sex. Focusing on bifenthrin, pyraclostrobin, topotecan, and (+/−) JQ1, we found that these chemicals induced expression effects across all concentrations in a highly concordant manner across sexes (Fig. 5). For example, the expression changes following bifenthrin exposure were exclusive to the 10 µM concentration in both male and female neurons, whereas pyraclostrobin altered gene expression similarly across all concentrations regardless of sex. Though there were some subtle differences in expression following exposure to topotecan, but not other topoisomerase inhibitors, across sexes that may warrant further exploration, our data suggest that chemical and concentration are the biggest determinants of a gene expression response. Future studies will statistically model concentration-dependent effects for each chemical, similar to work done by the United States Environmental Protection Agency^30^, and quantitatively compare those responses across sexes in a robust manner.

## Discussion

Our study provides an approach to prospectively identify environmental chemicals that transcriptionally mimic autism and other brain disorders in a rapid and cost-effective manner. We showed that the transcriptional signature most closely tied to autism and neurodegeneration is induced by chemicals that are highly related in terms of structure and/or their targeting of the mitochondrial electron transport chain. Our data now also implicate the related chloroplast photosystem I, which is inhibited by diquat dibromide^25^ and linked to Parkinson’s disease^10^. These findings suggest a convergence on the disruption of mitochondrial electron transport that may occur through multiple mechanisms, each resulting in transcriptional changes that mimic autism and neurodegeneration. An association between electron transport, autism, and Parkinson’s disease has been described previously^31–33^.

By assaying 56 genes, we reduce the search space and can rapidly identify new chemicals or mixtures that act similarly to Cluster 1 (primarily comprised of pyrethroids), Cluster 2 (primarily electron transport inhibitors), and Cluster 5 (topoisomerase inhibitors), as well as identify potentially novel chemical signatures (e.g. (+/−) JQ1). Transcriptomics studies that typically took months or years to complete can not only be reproduced but also expanded after just hours or days worth of sample and data processing using a targeted approach such as RASL-seq. It is worth noting, however, that the genes assayed may preclude the detection of novel transcriptional signatures where the effects are independent of the particular panel we assembled here. Moreover, chemical treatments that induce shifts in cell proportions within our neuronal cultures are difficult to resolve, though we previously demonstrated that the transcriptional changes associated with Cluster 2 chemicals occurred while proportions remained stable^9^.

In summary, our study represents a proof-of-principle that targeted transcriptomics enables high-throughput screening of large and diverse chemical libraries. There are more than 80,000 chemicals registered for use in the environment, and although we assayed <1% of them here, this approach represents the first step in identifying those that may carry the potential to harm the adult or developing brain^34^. Future studies using additional chemicals and concentrations to statistically model concentration- and sex-dependence can help predict neurotoxicity *in vivo*, thus providing a platform to prioritize chemicals and chemical classes for costly and time-consuming *in vivo* developmental and adult neurotoxicity testing.

## Methods

### Cortical neuron cultures

All animal experiments were approved by the Institutional Animal Care and Use Committee of the University of North Carolina at Chapel Hill and in accordance with NIH guidelines. Primary mouse cortical neuron cultures were prepared as previously described from E14.5 pregnant C57BL/6 J dams. The embryos were sexed using the REDExtract-N-Amp™ PCR ReadyMix™ kit (Sigma-Aldrich) for *Sry*^35^. Dissociated cells were plated in 384-well plates coated with poly-D-lysine (0.1 mg/ml) at a density of 20,000 cells/well in Neurobasal medium (Life Technologies) containing 5% fetal bovine serum (Invitrogen), B27 (17504-044, Invitrogen), Antibiotic-Antimycotic (15240-062, Invitrogen) and GlutaMAX (35050-061, Invitrogen). On days *in vitro* (DIV) 3, a full medium change was performed with feeding medium identical to the plating medium except that we omitted fetal bovine serum and included 5 μg/ml 5-fluoro-2′-deoxyuridine (F0503, Sigma-Aldrich) to inhibit mitosis in dividing cells. Cells were also plated into tissue culture plates and treated in the same way as the cells in 384-well plates to generate conditioned media to be used during dosing.

### Drug dosing

Drug dosing was done using a Tecan EVO liquid handling robot. On DIV 7, a full media change was performed in two steps. Step 1: 15 µL of conditioned media was added to the cells. Step 2: 4X concentration drugs were diluted in 5 µL of conditioned media and was added to the cells to give a total 1X concentration of the drug in 20 µL of the media. The final concentration of DMSO in every sample was at 0.1%. The vehicle controls carried only 0.1% DMSO and no drug. The neurons were dosed with the respective drugs for 24 h at 37 °C before lysing. A total of 294 ToxCast Phase I chemicals, 54 other commonly used chemicals, chemical mixtures, and controls at four concentrations per sex, in biological triplicate, were assayed; certain chemicals of interest were also assayed multiple times (Supplementary Data S5).

### Cell lysing

Prior to lysing, cells were washed once with 20 µL PBS, left on the cells for 1 minute. PBS was removed, then 1X Nucleic Acid Purification (NAP) lysis solution diluted in PBS was added. The lysate was mixed gently by pipetting up and down to assist in the lysing process. The plates were immediately stored at −80 °C.

### Preparation of Oligo (dT) Magnetic Beads

Oligo d(T) magnetic beads were washed twice in Binding and Washing buffer. Beads were further washed with 0.2% SDS, followed by a wash with 4X SSC buffer. The beads were finally resuspended in 4X SSC solution before addition to the cell lysate.

### RASL probes

Multiple probes (3–7) were designed per gene for most genes assayed. The probes were designed to span exon junctions when possible and amplify different regions across respective genes. Pilot studies identified probes that consistently worked, giving a total of 261 probes for 56 genes.

### Pooling Probes

Probes were pooled, and diluted in water to a final concentration of 20 nM. A luciferase spike-in mRNA (Promega Corporation, catalog number L4561) was added to the probe pool at a final concentration of 1 nM. (Supplementary Data S1).

### Barcoding primers master plates

A final concentration of 2 µM of forward and reverse primers master plates were used for every reaction. 4.5 µL of each were added to every RASL reaction. (Supplementary Data S2).

### RASL reaction

5 µL of 4X oligo (dT) magnetic bead solution and 10 µL of lysate were added to every well containing 5 µL of 4X probe pool and was mixed thoroughly by pipetting. The plate was sealed and incubated at 60 °C for 10 min following 45 °C for 30 minutes. Beads were immediately collected on a magnet and the supernatant was removed from each well. The beads were then re-suspended in 20 µL of 1X ligase buffer. The plate was sealed and incubated at 45 °C for 2 min. The beads were collected on a magnet and buffer supernatant was removed. The beads were re-suspended in 20 µL of 1X ligase buffer containing 2 U of Rnl2. The plate was sealed and incubated at 37 °C for 30 min.

Beads were immediately collected on a magnet and the supernatant was removed from each well. The beads were re-suspended in 6 µL of Omni Klentaq PCR master mix containing dNTPs and 2 U polymerase. 4.5 µL each of forward and reverse primers we added next making it a total PCR volume of 15 µL. The plate was placed in the thermocycler for PCR (Thermocycling: 95 °C for 2 min; X2 Cycles 95 °C for 20 s and 52 °C for 30 s; X27 Cycles 95 °C for 20 s and 60 °C for 30 s; 4 °C hold forever). The plate was stored at −20 °C until amplicon pooling. PCR product (3 µL) from every well was pooled into a single pool of barcoded PCR products. Samples were further concentrated using a PCR Cleanup kit. The expected 181 bp PCR bands were gel purified. The concentrations were determined using Nanodrop. The sample concentrations for every plate were normalized and pooled together such that every plate was equally represented in the pool. The samples can be stored at minus 20 °C.

### High-throughput sequencing

A total of three biological replicates were processed independently. Each biological replicate consisted of 384-well plates (four male and four female, for a total of eight plates), with a sample size of 3,072. The 9,216 samples from the three replicates were pooled together and data was obtained in a single sequencing run, therefore eliminating a major source of batch effects. The RASL pool was sequenced using two lanes of an Illumina HiSeq 2500 Rapid Run.

### RASL-seq data processing

Raw counts per probe were obtained using CLC Genomics Workbench. Counts for luciferase spike-in probes were separated, then the remaining probes and wells were filtered such that the total number of reads for a given well exceeded 500, and the total number of reads per probe exceeded 1000 (summed across all 9,216 samples). This removed poorly amplified probes or wells. A psuedocount of 1 was added to all values, then the geometric mean of control probes (*Ascl3*, *Psmd4*, *Sdha*, *Tbp*) was computed for each well. Each value was then scaled by taking the log_2_ ratio relative to the geometric mean of controls. Replicate probes were then collapsed by gene symbol by taking the median, resulting in 2,791 chemical-concentration-sexes that passed quality control filters. Expression values for each gene were then normalized using a median-polish procedure, and biological (well) replicates were collapsed by taking the median. For clustering, expression values for *Ddx3y* and *Xist* were separated, then expression values were median-centered and clustered hierarchically. Sex of the neurons was known based on genotyping (see above), and was confirmed by subtracting *Ddx3y* from *Xist* expression. Cell health was estimated by computing the fraction of total reads in a given well corresponding to luciferase spike-in probes.

### Comparisons to previous expression datasets

Each of the six previously identified clusters^9^ contained multiple chemicals, so to enable a more direct comparison, we collapsed these down into six aggregate expression signatures by taking the median of normalized gene expression values across chemicals for each gene. For each chemical-concentration-sex, we then computed Pearson correlations to each of these six signatures for just the set of genes commonly assayed by RNA-seq and RASL-seq. To refine our previous cluster assignments and assess the role of dosage, we retained only those chemicals for which at least one concentration correlated with at least one of the six signatures above a correlation threshold of 0.65. No such chemicals remained for Clusters 3, 4, or 6, suggesting that these expression changes could not be reproduced by RASL-seq, possibly because of the marker genes used and/or because we previously used F_1_ hybrid C57BL/6 × CASTEi/J neuron cultures^9^. Numerous chemicals and concentrations remained, however, for Clusters 1, 2, and 5. Two chemicals correlated with more than one cluster, namely bardoxalone-methyl and triphenyltin hydroxide. Since these chemicals were among the worst cell health scores (Supplementary Data S6) and could not be resolved to just one transcriptional signature or cluster, we omitted them from subsequent analyses and refer to them as “unclassified”.

## Supplementary information

Supplementary Info

Dataset 1

Dataset 2

Dataset 3

Dataset 4

Dataset 5

Dataset 6

Dataset 7

## Data Availability

All data generated or analyzed during this study are included in this published article (and its Supplementary Information files). We have also utilized a web-based interactive tool for data visualization; our normalized data as presented in Fig. 2 can be viewed at: http://zylkalab.org/data.
