# Supplementary material for: High-throughput screening and classification of chemicals and their effects on neuronal gene expression using RASL-seq
Source: Sci Rep. 2019 Mar 14;9:4529. doi: 10.1038/s41598-019-39016-5 (PMC6418307; doi:10.1038/s41598-019-39016-5)
Supplement: Supplementary file 1 — Supplementary Info [file 41598_2019_39016_MOESM1_ESM.pdf]

Supplementary Information

High-throughput screening and classification of chemicals and their effects on neuronal gene expression using RASL-seq

Jeremy M. Simon, Smita R. Paranjape, Justin M. Wolter, Gabriela Salazar, Mark J. Zylka

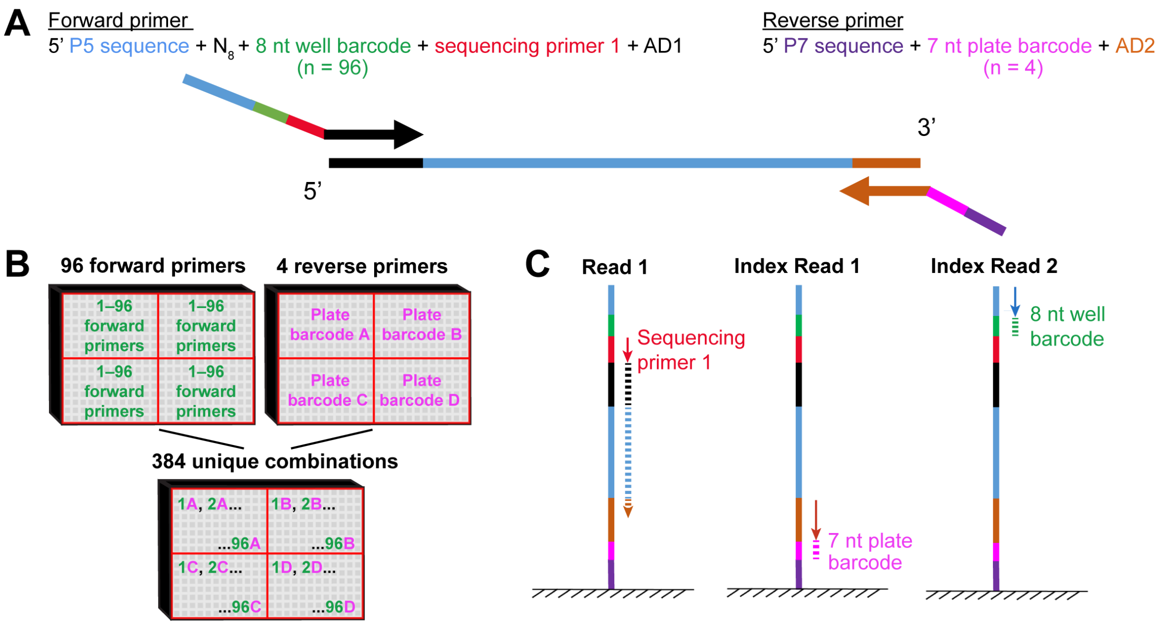

Supplementary Fig. S1. RASL-seq primer and plate design. A. Primer design for RASL-seq probes. **B.** Plate setup for forward-reverse primer combinations. **C.** High-throughput sequencing of barcoded amplicons.

A

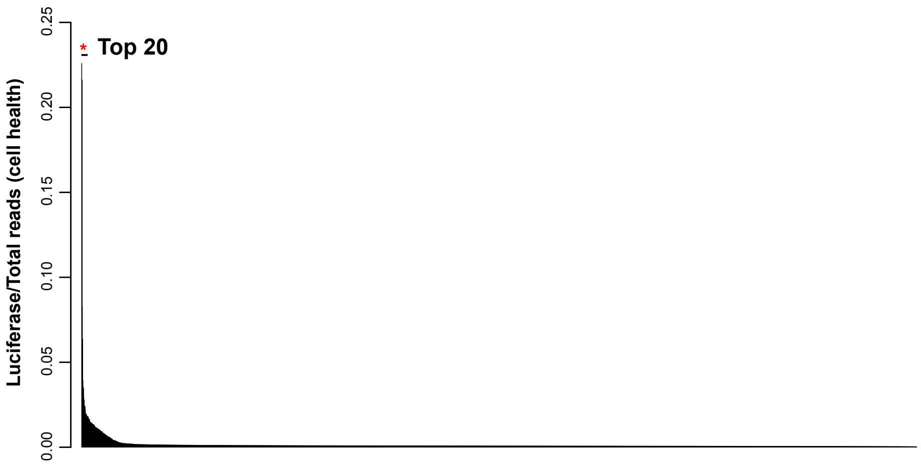

B

Chemical	Concentration (μM)	Sex	Cluster Assignment	Chemical Type	Cell Health Score
Triphenyltin hydroxide	10	Female	Unclassified	Fungicide	0.226
Emamectin benzoate	10	Female	Unclassified	Insecticide	0.216
Bardoxalone-methyl	10	Female	Unclassified	NRF2 activator	0.083
Azoxystrobin + Chlorothalonil	10	Male	Unclassified	Mitochondrial inhibitor + Fungicide	0.064
Fluazinam	10	Male	Cluster 2	Fungicide	0.039
Trifloxystrobin	10	Male	Cluster 2	Fungicide	0.036
Pyraclostrobin + Boscalid	0.1	Female	Cluster 2	Mitochondrial inhibitor + Fungicide	0.035
Triphenyltin hydroxide	1	Female	Unclassified	Fungicide	0.029
Azoxystrobin + Chlorothalonil	10	Female	Unclassified	Mitochondrial inhibitor + Fungicide	0.028
Fluazinam	10	Female	Cluster 2	Fungicide	0.025
Abamectin	10	Male	Unclassified	Insecticide	0.024
Kresoxim methyl + Boscalid	10	Male	Cluster 2	Mitochondrial inhibitor + Fungicide	0.024
Pyridaben	0.01	Female	Cluster 2	Insecticide	0.022
Pyraclostrobin + Boscalid	1	Female	Cluster 2	Mitochondrial inhibitor + Fungicide	0.020
Trifloxystrobin	10	Female	Cluster 2	Fungicide	0.020
Rotenone	0.1	Female	Cluster 2	Insecticide	0.019
Rotenone	1	Female	Cluster 2	Insecticide	0.019
Sodium arsenite	10	Female	Unclassified	Herbicide	0.018
Fenpyroximate (Z,E)	0.1	Male	Cluster 2	Insecticide	0.018
Rotenone	0.1	Male	Cluster 2	Insecticide	0.018

Supplementary Fig. S2. Cell health scores. A. Cell health was computed by quantifying the luciferase spike-in values relative to the total read count per well and plotted as a barplot in ranked order. **B.** The top 20 cell health scores by chemical-concentration-sex are tabulated along with chemical cluster annotations. Since bardoxalone-methyl and triphenyltin hydroxide were associated with more than one cluster, they are listed as “unclassified”.

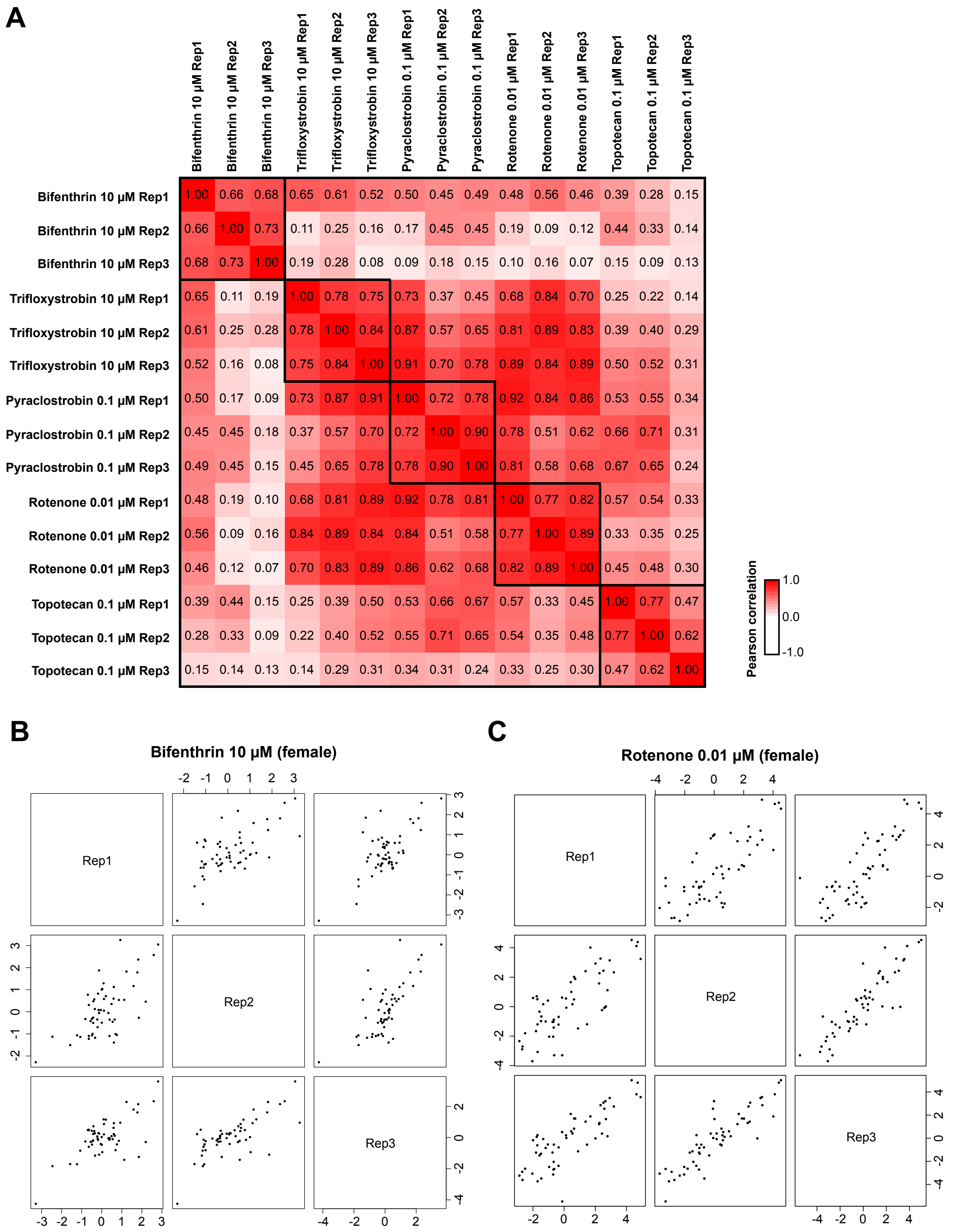

Supplementary Fig. S3. Cross-replicate RASL-seq concordance. A. Pairwise Pearson correlations for representative chemicals, for female neurons. Normalized expression values were used to compute the correlations. **B–C.** Scatterplots of normalized expression values illustrating concordance among triplicates pairwise. Each point is a gene; all 56 genes are plotted.

Plate	Set 1 (%)	Set 2 (%)	Set 3 (%)
**0.01 µM Male**	98.42	98.3	96.75
**0.1 µM Male**	98.54	97.26	97
**1 µM Male**	98.16	97.26	93.57
**10 µM Male**	91.09	97.34	98.54
**0.01 µM Female**	99.02	97.94	99.27
**0.1 µM Female**	99.1	98.22	93.85
**1 µM Female**	99.22	93.35	96.77
**10 µM Female**	99.23	96.93	99.26

Supplementary Table S1. Percentage of correctly mapped reads for each biological replicate, separated by concentration and sex.

Supplementary Data S1. RASL-seq primer sequences and characteristics.

Supplementary Data S2. Barcoded RASL-seq primer sequences.

Supplementary Data S3. List of chemicals and chemical categories interrogated by RASL-seq.

Supplementary Data S4. RASL-seq probe settings.

Supplementary Data S5. Raw read counts table for all RASL-seq probes and wells. Data is provided for all three biological replicates, for all wells and probes prior to quality filtering and normalization.

Supplementary Data S6. Chemical categorization and cell health scores. Cell health was computed by quantifying the luciferase spike-in values relative to the total read count per well. The associations with chemical clusters is also shown here; since bardoxalone-methyl and triphenyltin hydroxide were associated with more than one cluster, they are listed as “unclassified”.

Supplementary Data S7. Normalized data table for all RASL-seq probes and wells that passed filtering criteria. Data were filtered, scaled by control probes, median-polished, and biological replicates were combined as described. Hierarchical clustering was performed on these data, but *Xist* and *Ddx3y* were first removed, and data were median-centered.
